# The value of computed tomography-based radiomics for predicting malignant pleural effusions

**DOI:** 10.3389/fonc.2024.1419343

**Published:** 2024-08-12

**Authors:** Zhen-Chuan Xing, Hua-Zheng Guo, Zi-Liang Hou, Hong-Xia Zhang, Shuai Zhang

**Affiliations:** ^1^ Department of Pulmonary and Critical Care Medicine, Beijing Luhe Hospital, Capital Medical University, Beijing, China; ^2^ Department of Infectious Diseases, Beijing Luhe Hospital, Capital Medical University, Beijing, China

**Keywords:** radiomics, pleural effusion, x-ray computed tomography, cancer, machine learning

## Abstract

**Background:**

Malignant pleural effusion (MPE) is a common clinical problem that requires cytological and/or histological confirmation obtained by invasive examination to establish a definitive diagnosis. Radiomics is rapidly evolving and can provide a non-invasive tool to identify MPE.

**Objectives:**

We aimed to develop a model based on radiomic features extracted from unenhanced chest computed tomography (CT) images and investigate its value in predicting MPE.

**Method:**

This retrospective study included patients with pleural effusions between January 2016 and June 2020. All patients underwent a chest CT scanning and medical thoracoscopy after artificial pneumothorax. Cases were divided into a training cohort and a test cohort for modelling and verifying respectively. The Mann-Whitney U test and the least absolute shrinkage and selection operator (LASSO) were applied to determine the optimal features. We built a radiomics model based on support vector machines (SVM) and evaluated its performance using ROC and calibration curve analysis.

**Results:**

Twenty-nine patients with MPE and fifty-two patients with non-MPE were enrolled. A total of 944 radiomic features were quantitatively extracted from each sample and reduced to 14 features for modeling after selection. The AUC of the radiomics model was 0.96 (95% CI: 0.912-0.999) and 0.86 (95% CI: 0.657~1.000) in the training and test cohorts, respectively. The calibration curves for model were in good agreement between predicted and actual data.

**Conclusions:**

The radiomics model based on unenhanced chest CT has good performance for predicting MPE and may provide a powerful tool for doctors in clinical decision-making.

## 1 Introduction

Malignant pleural effusion (MPE) is a common medical problem caused by both primary and secondary pleural malignancies. Mesothelioma is the predominant primary pleural malignancy, which is associated with a history of asbestos exposure and is a rare tumor. Secondary malignant pleural effusions are mainly caused by pleural metastases from lung and breast cancer and account for 50-65% of malignant pleural effusions (1).

Epidemiological information is limited, but there are an estimated 50,000 new diagnoses of MPE in the UK each year and over 125,000 hospitalized patients with MPE in the United States per year (2, 3). It is expected that there will be approximately 4,820,000 new cancer cases in China in 2022 and the main types of cancer will be lung, colorectal, stomach, liver and breast cancers (4). An increasing number of cancer cases leads to a higher incidence of MPE. The presence of MPE indicates that the tumor has spread or progressed to advanced stages, and the median survival of patients is only 3 to 12 months from the time of diagnosis (5). Selecting the optimal management strategy to minimize invasiveness and discomfort in patients with advanced cancer, correct identification of MPE is necessary.

A definitive diagnosis of MPE requires cytological and/or histological confirmation (2). Therefore, in patients with suspected MPE, various invasive techniques have to be used in order to obtain sufficient tissue samples to definitively make the diagnosis (1).. Unfortunately, not all patients are fit to undergo the procedure due to complications. In this situation, diagnostic workup relies heavily on medical history and imaging. Chest CT is the primary imaging test for MPE, which can show abnormal changes in the pleura and provides a reliable basis for diagnosis. The presence of nodular pleural thickening, mediastinal pleural thickening, parietal pleural thickening > 1 cm, and circumferential pleural thickening in CT images are supportive of malignant diseases (2). However, in clinical practice, even if CT is interpreted by 2 experienced radiologists, one in three patients with MPE is still missed (6). Radiomics is a sophisticated image analysis technology with rapid development. It helps to improve diagnosis and can provide a powerful tool for modern medicine by extracting quantitative medical image features and capturing the characteristics of tissues and lesions (7–9). Herein, we aimed to identify patients with MPE by CT image radiomics analysis.

## 2 Methods

### 2.1 Study population

This study was approved by the Ethical Committee of Beijing Luhe Hospital, Capital Medical University (2021-LHKY-094-02). All patients provided written informed consent. The inclusion criteria were as follows: (1) malignant pleural effusion patients were confirmed by pathological examination; (2) nonmalignant pleural effusion cases were followed up for 1~2 years and no malignant changes occurred; and (3) complete chest CT images after artificial induced pneumothorax. The exclusion criteria were as follows: (1) low-quality or incomplete CT images after artificially induced pneumothorax; and (2) para malignant pleural effusions. A total of 29 patients with MPE (12 males and 17 females; age range, 26-83 years; mean age, 63.14 ± 13.45 years) and 52 patients without MPE (38 males and 14 females; age range, 18-85 years; mean age, 52.98 ± 20.13 years) in the respiratory ward of our hospital between January 2016 and June 2020 were included. All cases were divided into a training cohort and a test cohort using the stratified random resampling method at a ratio of 4:1. The training cohort was used to tune the parameters and develop the prediction model, while the test cohort was utilized to evaluate the predictive performance of the model. All clinical results were extracted from the patients’ electronic medical records in the hospital information system.

### 2.2 CT scanning

All patients were evaluated and signed informed consent forms before examination. Artificially induced pneumothorax was formed by injecting filtered air into the affected thoracic cavity. The induced pneumothorax volume was 300 ml. During CT scanning, the patient maintained a healthy lateral position. All CT scans were obtained by using a Philips 16-slice spiral CT machine. Scanning parameters: tube voltage 80 kV, tube current Auto mA, matrix 512 × 512, rotation time 0.5 s, pitch 0.938 mm, slice thickness of 5 mm, and slice spacing of 5 mm. The scanning scope covered the whole lung field.

### 2.3 Radiomics workflow

**Figure 1:**
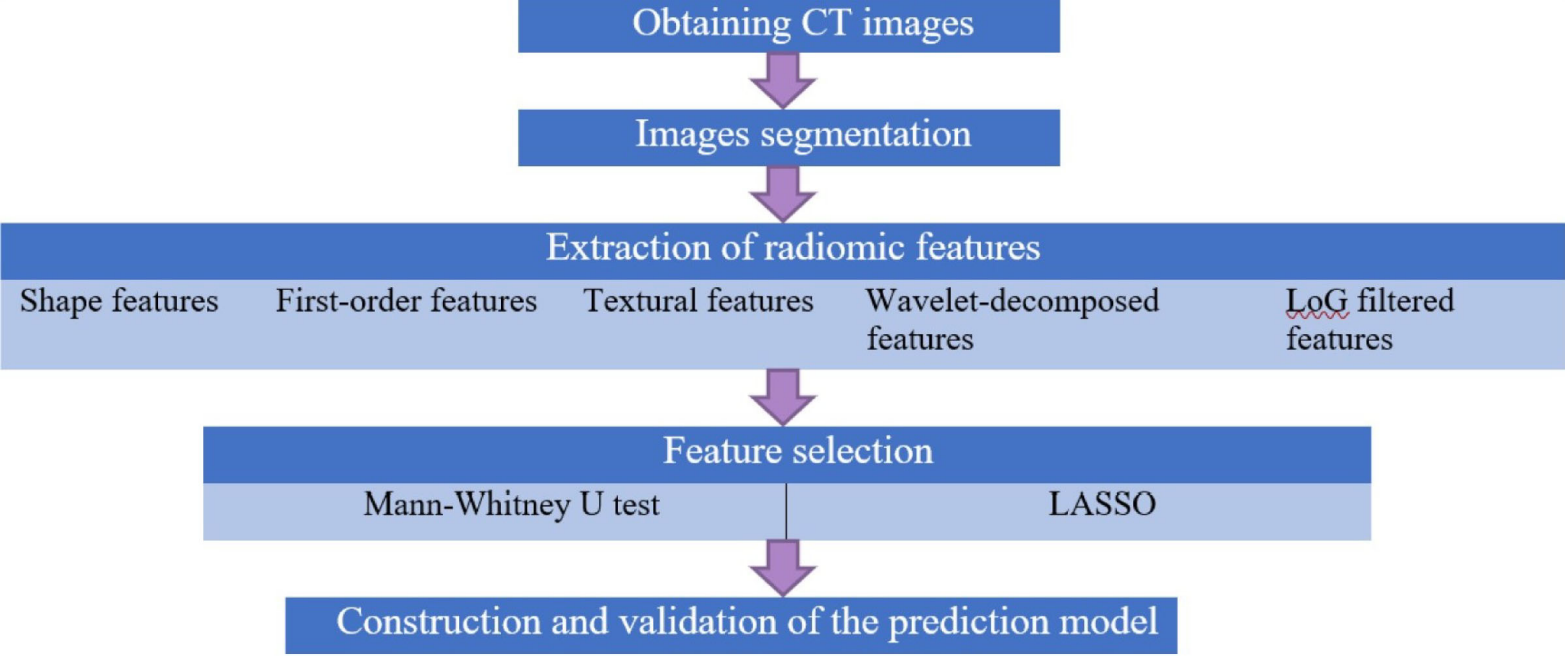
Flow diagram of radiomics analysis in these analyses. Core steps of radiomics analysis.

The core steps of radiomics analysis included ROI segmentation, feature extraction, feature selection and predictive model building (shown in **Figure 1**).

#### 2.3.1 ROI segmentation

**Figure 2:**
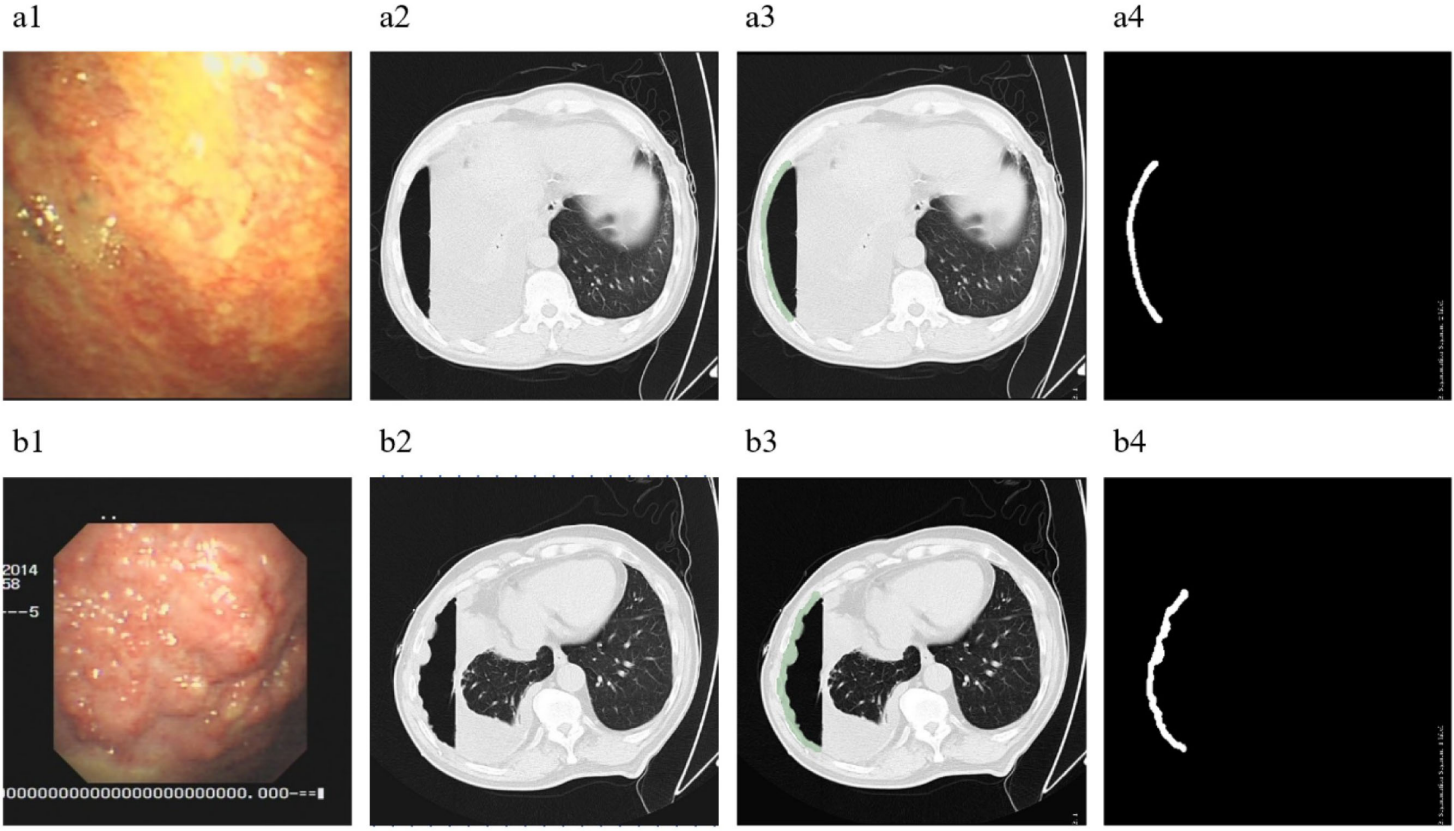
ROI segmentation. **(A)**, A 62-year-old male with PE, diagnosed as tuberculous pleurisy. **(B)**, A 67-year-old male with PE, diagnosed as malignant pleural mesothelioma. 1, Images of pleural lesions under medical thoracoscopy. 2, CT images of patients after artificial pneumothorax. 3, Regions of interest segmented on the CT under lung window. 4, The regions of interest obtained after segmentation.

We used the open-source software 3D-slicer (version 4.11, http://www.slicer.org) as the analysis platform. All visible layers of wall pleura exposed to pneumothorax were selected as the regions of interest (ROI), as it is difficult to accurately identify localized diffuse pleural lesions with the naked eye. Regions of interest were manually delineated slice-by-slice by an experienced respiratory physician and a radiologist on lung window background (level, −500 HU; width, 1,300 HU) axial CT images (shown in **Figure 2**). Consensus was reached by discussion in case of disagreement.

#### 2.3.2 Radiomic feature extraction and selection

All images were normalized before extraction. We used the in-house software “pyradiomics” package based on Python (version 3.7.1, http://www.python.org) to extract radiomic features from ROIs. Meaningless data in the list of radiomic features were deleted manually. A total of 944 high-dimensional features were extracted from each patient. The features consisted of four categories: (a) shape features: n = 14; (b) first-order statistical features: n = 18; (c) textural features derived from texture matrices including gray-level cooccurrence matrix (GLCM), gray-level dependence matrix (GLDM), gray-level run length matrix (GLRLM), gray-level size zone matrix (GLSZM), neighborhood gray tone difference matrix (NGTDM): n=24 + 14 + 16 + 16 + 5 = 75; and (d) transformed features: wavelet-decomposed features in frequency channels LHL, LLH, HHH, HLH, HLL, HHL, LHH and LLL: n=[18 + 75]×8 = 744; Laplacian of Gaussian (LoG) filtered features: n=18 + 75 = 93. Feature selection comprised two steps: the Mann-Whitney U test and the least absolute shrinkage and selection operator (LASSO) method. The optimized hyperparameter λ was settled using 10-fold cross-validation and the robust and nonredundant features were selected based on the determined optimal λ. Finally, we obtained 14 optimum features.

#### 2.3.3 Building the radiomics model

We developed a radiomics model using the support vector machine (SVM). The radial basis function (RBF) was applied to solve the nonlinear problem, and GridSerchCV was used to find the best (C, g) for the kernel, where ‘C’ is the cost parameter and ‘g’ is the coefficient parameter in the RBF.

### 2.4 Evaluation of model performance and statistical analysis

The discriminatory performance of the radiomics model was evaluated by receiver operating characteristic (ROC) analysis. The area under the curve (AUC), accuracy, sensitivity and specificity were calculated in both the training cohort and test cohort. The calibration curves and Hosmer and Lemeshow test were plotted to assess the goodness-of-fit of the model. All statistical analyses were performed by using SPSS (version 25.0). Continuous variables with a normal distribution are illustrated as the mean ± standard deviation. The reported statistical significance levels were all two sided, with statistical significance set at.05.

## 3 Results

### 3.1 General information of patients

A total of 81 patients were included in this study, of whom 29 (35.80%) were diagnosed with MPE and 52 (64.20%) were diagnosed with non-MPE. The 29 patients with MPE included 4 (13.79%) cases of malignant pleural mesothelioma, 1 (3.45%) case of malignant lymphoma and 24 (82.76%) cases of metastatic tumor of the pleura (19 lung adenocarcinoma, 3 breast cancer, 1 lung squamous cell carcinoma, 1 gastric cancer). The 52 patients with non-MPE included 27 cases (51.92%) of parapneumonic effusions, 24 (46.15%) cases of tuberculous pleural effusions, and 1 (1.92%) case of pleural empyema.

### 3.2 Radiomics analysis

**Figure 3:**
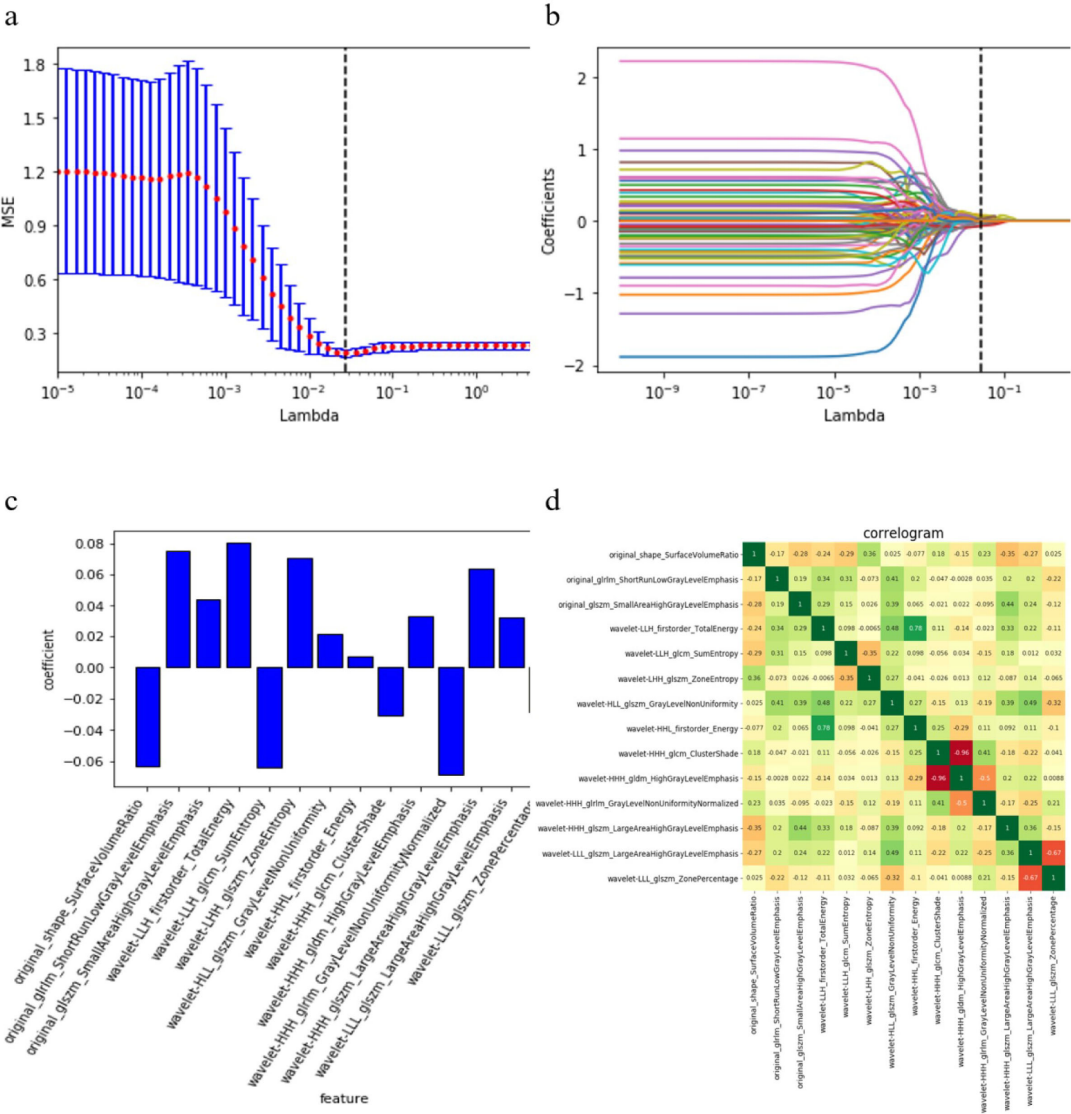
Fourteen radiomics features selected. **(A)**, Adjusting the parameter λ to minimize the binomial deviation of the model fitting loss value, in order to select the best radiomics characteristics. **(B)**, The distribution of LASSO coefficients of radiomics. **(C)**, The weights of features contributed in the model built. **(D)**, The correlation heatmap of the fourteen features selected.

Following the image-preprocessing procedure, 944 unenhanced CT radiomic features were extracted from each sample. One hundred ninety-two features were selected using the Mann-Whitney U test, while 14 effective predictors with nonzero coefficients were chosen after LASSO (shown in **Figures 3A, B**). The corresponding coefficients were evaluated (shown in **Figure 3C**). Heatmap of fourteen features are shown in **Figure 3D**. The prediction model was constructed using SVM. We set the range of C as [2^–2^, 2^4^] and the range of g as [–3, 1] and selected the optimal mode parameters (C, g) as (2.777, 0.018) using GridSerch. Subsequently, a radiomics model was created.

### 3.3 Performance of the radiomics model

**Figure 4:**
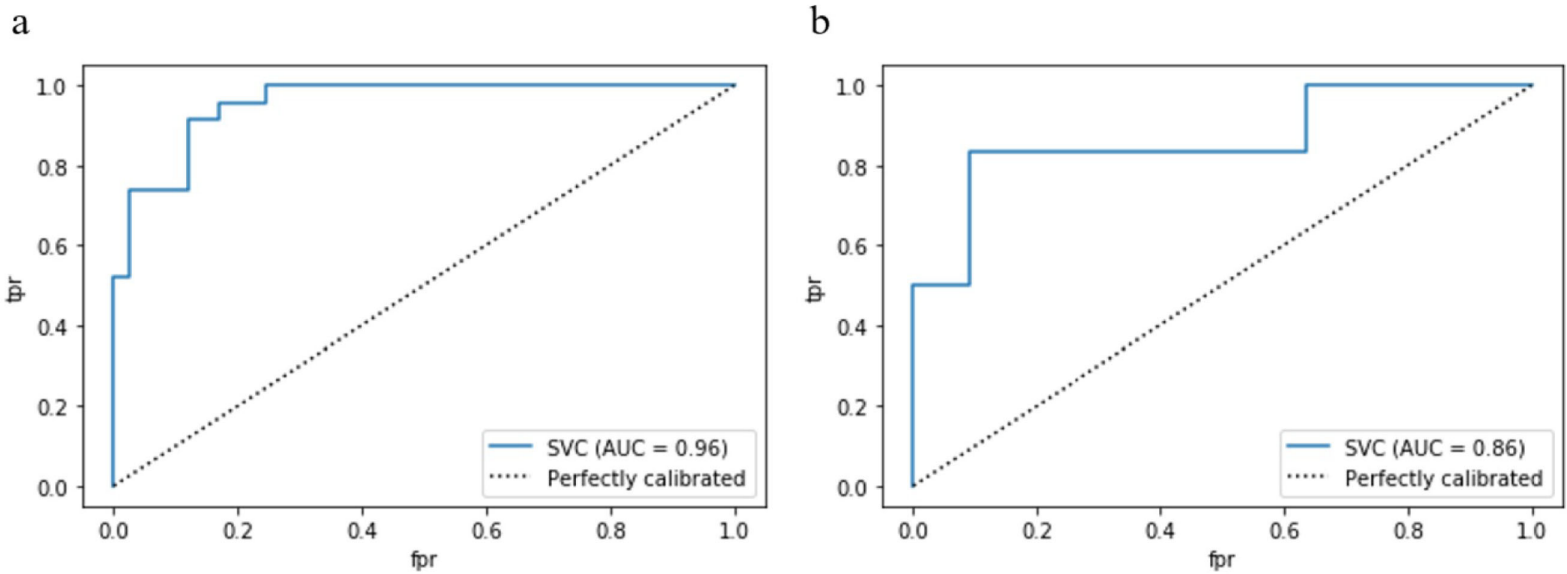
The receiver operator characteristic (ROC) curves of the radiomics model in the training cohort **(A)** and test cohort **(B)**. AUC, area under the receiver operator characteristic curve. .

**Figure 5:**
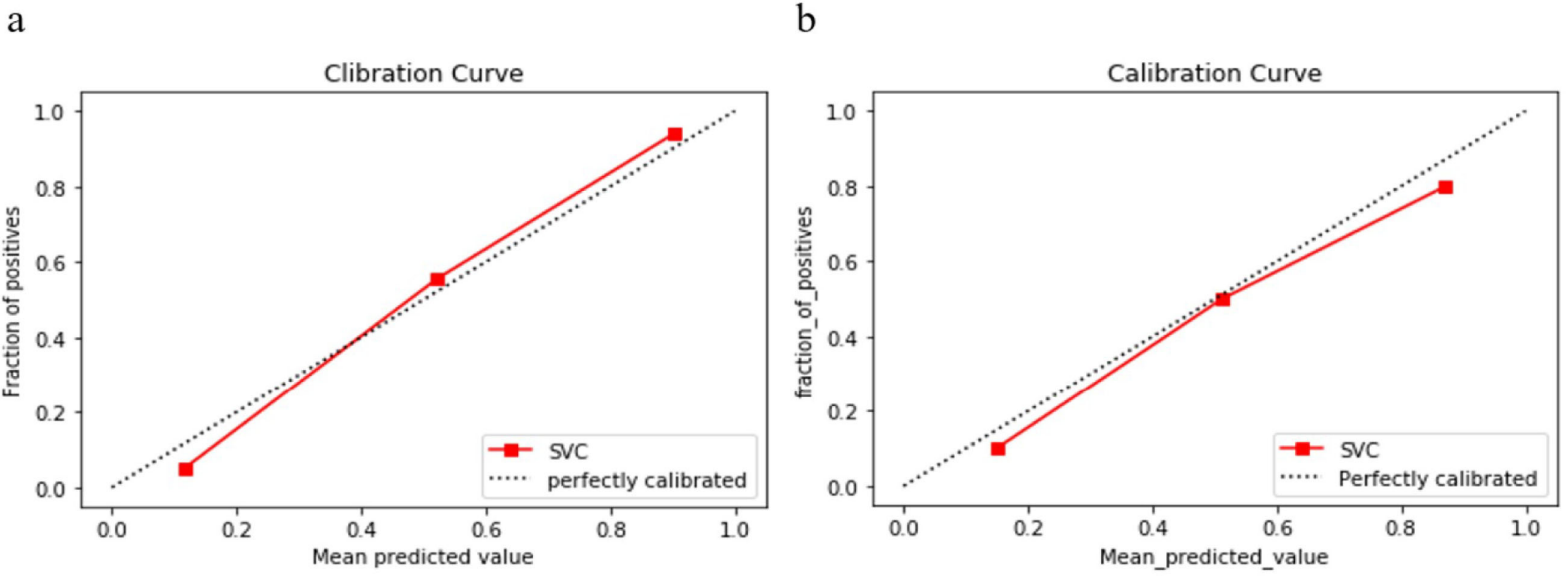
Calibration curves of the radiomics model in the training cohort **(A)** and test cohort **(B)**. The y axis represents the actual event probability, the x axis represents the predicted event probability. The 45° dotted line represents the perfect prediction of an ideal model and the dotted lines represents the performance of the radiomics model, a closer fit to the dotted line represents a better prediction. The calibration curves indicate good calibration of the model in the training and test cohorts.

The radiomics model yielded AUCs of 0.96 (95% CI: 0.912-0.999) and 0.86 (95% CI: 0.657~1.000) in the training and test cohorts, respectively (shown in **Figure 4**). The accuracy, sensitivity and specificity of the model for the training and test cohorts are shown in **Table 1**. The calibration curve of the model demonstrated good agreement between the predicted and observed MPE in the training and test cohorts (shown in **Figure 5**). The Hosmer–Lemeshow test yielded nonsignificant differences (P> 0.05).

**Table 1:** Predictive and diagnostic values of radiomics model.

	AUC (95% CI)	ACC	SEN	SPE	PPV	NPV
Training cohort	0.96 (0.912, 0.999)	0.891	0.739	0.976	0.944	0.870
Test cohort	0.86 (0.657, 1.000)	0.882	0.833	0.909	0.833	0.909

AUC, area under the receiver operating characteristic curve; CI, confidence interval. ACC, accuracy; SEN, Sensitivity; SPE, sensitivity; PPV, positive predictive value; NPV, negative predictive value.

## 4 Discussion

In this study, we developed a model based on radiomic features extracted from unenhanced chest CT for the noninvasive prediction of MPE. The model performed satisfactorily in both the training cohort (AUC, 0.96) and the test cohort (AUC, 0.86) and had good calibration. The results demonstrated that radiomics model can be a reliably tool to help clinically predict MPE.

It is a challenge to establish a diagnosis of MPE on the basis of minimizing invasiveness and discomfort for the patient. CT imaging is one of the most valuable noninvasive tests for patients with suspected MPE and is widely used in clinical practice. CT-based identification of malignant pleural disease or malignant pleural effusion has a sensitivity of 65% to 72% and a specificity of 78% to 98% (10–12). As shown above, the sensitivity of conventional radiographic assessment of tumors in identifying malignant pleural disease is unsatisfactory, which is probably related to its heavy reliance on visual interpretation. Radiomics enables digital decoding of radiographic images into quantitative features, allowing extraction of more detailed characteristics, even information that is undetectable by humans (8). It combines image processing and data mining techniques and is a new interdisciplinary discipline used to solve medical problems. A great deal of research has been carried out in the diagnosis of malignant tumors such as lung, breast, and prostate cancers based on radiomics, and has shown great potential and broad application prospects. The main causes of MPE are lung malignancies, breast carcinoma and malignant mesothelioma, and our study is consistent with this finding (1, 5). There are few studies on the identification of MPE via radiomics methods and we retrieved only 1 relevant article. This study built a model based on CT images of 315 patients with pleural effusion. The AUC of the model was 0.876 and 0.774 in the training and test groups, respectively (13). Even though the research conducted in the area of imaging-based radiomics to identify MPEs is inadequate, extensive studies have been conducted on the diseases that most commonly lead to MPEs, such as lung cancer and breast cancer. These studies have shown that CT-based radiomics performs well in identifying malignant lesions in the lungs (14–18) and in recognizing dry pleural spread in non-small cell lung cancer and visceral pleural invasion in lung adenocarcinoma (19–21). Recent studies have also shown the great potential of radiomics in differentiating benign and malignant breast lesions (22).MPM is the main primary malignant tumor causing MPE, and genomic studies have shown that MPM is dominated by inactivation of tumor-suppressor genes. Up to 70% of MPM patients have mutations in the BAP1 and CDKN2A genes (23). Studies associated with the recognition of MPM based on radiomics are lacking. While, a study by Liu Lei et al. (24) showed that a 3D radiomic model based on unenhanced CT imaging has good predictive performance for BAP1 mutation status in MPM. Therefore, we believe that radiomics might be helpful in the clinical diagnosis of mesothelioma. Based on these findings, we conducted a preliminary study on the value of predicting MPEs based on radiomics. Encouragingly, the findings are promising. Our study demonstrates that radiomics exhibits superior sensitivity and good specificity in predicting MPE compared to conventional CT assessment. Our study has several limitations. First, it was a single-center retrospective study with possible selection bias. Second, the sample size included in the study was small. Third, the radiomics model we developed has not yet been externally validated, and its generalizability and applicability need to be further verified. Nonetheless, this preliminary study still suggest that CT-based radiomics could be useful in predicting MPE. It will provide clinicians with a noninvasive way to narrow down the differential diagnosis in the first place, and if malignant exudates are suggested using our model, we will do our best to find a basis of malignant exudates. This area is worthy of further research. In the future, we will conduct studies with large sample sizes and combine radiomic features with clinical features to build deep learning models to obtain more credible evidence.

In summary, the radiomics model based on CT have initially shown good performance in predicting MPE and may hold promise in providing clinicians with a powerful noninvasive diagnostic tool to make more accurate decisions.

## Data Availability

The original contributions presented in the study are included in the article/supplementary material. Further inquiries can be directed to the corresponding author.
